# Unveiling Ionic/Electronic Contributions to the Potential Development of Electrical Double Layer Using XPS

**DOI:** 10.1021/acs.jpclett.5c01855

**Published:** 2025-08-20

**Authors:** Ezgi Kutbay, Burak Ulgut, Coskun Kocabas, Sefik Suzer

**Affiliations:** † Department of Chemistry, 52948 Bilkent University, 06800 Ankara, Türkiye; ‡ Materials Department, 5292 Manchester University, Manchester M13 9PL, United Kingdom

## Abstract

Time-dependent X-ray photoelectron spectroscopy (XPS) data are recorded for capturing dynamics of the electrical double layer (EDL) formation on two multilayered graphene electrodes, configured as a coplanar-capacitor, with an ionic liquid (IL) as the electrolyte. The device is subjected to 2 V biasing cycles, reversing its polarity every hour, while iteratively recording the O 1s peak representing the IL with 0.5 s steps, which turns XPS into a recording chemical voltmeter, since variations in binding energy position of the O 1s peak on electrodes report directly the local electrical potential of the IL medium. If the device is physically shorted during measurements, the potential suddenly jumps to the opposite polarization since this process nulls the electronic component of the potential, retaining the ionic one. Complementing action is captured on the drain electrode, which exhibits voltage decay(s) toward the system’s ground. On the other hand, data recorded when the circuit is temporarily opened reveal only decays without polarity reversal. Generally, these potentials capture decay with relatively long and multiple time constants, associated with ionic motion. The polarity reversal is explained by a simple model, considering the huge time constant difference between electronic and ionic motions.

## Graphical abstract

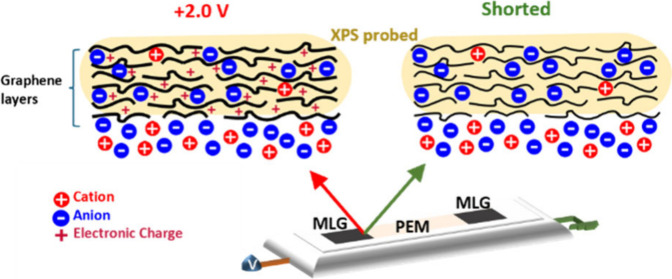

Electrical double layer (EDL) is a central theme for energy harvesting and storage systems, such as supercapacitors, where ionic liquid (IL) electrolytes are advocated as a favorable alternative, due mostly to their wider electrochemical windows, compared to aqueous and/or nonaqueous liquid electrolytes.^1–10^ As electrodes, carbonaceous materials, in numerous forms, such as graphite, graphene, carbon-black, etc., are the most studied ones, especially when they are produced as high surface area porous structures for charge storage and retrieval.^11–13^ The last three decades have witnessed a colossal and coordinated research effort to better understand, at the atomic and molecular level, the role of each and every component of these electrochemical systems, using state-of-the-art electrical, spectroscopic, and microscopic techniques.^14–22^ For example, a recent report using NMR measurements revealed a puzzling finding that more disordered carbonaceous materials with smaller graphene-like domains show higher capacitance, owing to more efficient ion storage.^23^ Experimental findings have also been corroborated with theoretical and simulation efforts.^24–27^ Recent simulations report that experimentally observed very long time constants can also be accounted for using the transmission-line approcah.^28–30^

The majority of electrochemical methods for elucidating dynamics at or around EDL rely on amperometric measurements,^18^ since techniques for complementing independent electrical potential measurements are limited. Moreover, NMR, optical, IR, Raman, etc. and combined spectro-electrochemical methods can only indirectly infer information about local electrical potential developments.^31–35^ This is where operando X-ray photoelectron spectroscopy (o-XPS) stands out, due to its superior chemical sensitivity and its unique ability to record local electrical potential evolution directly with temporal- and/or lateral-resolution, which has been reported by numerous researchers over the last five decades, as summarized in our recent review.^36^ In parallel, use of synchrotron facilities has tremendously enhanced our knowledge on the subject.^37,38^

More recently, o-XPS was used to record real-time local surface potentials at the electrode–electrolyte interfaces of a solid-state battery,^39^ similar to our reports on an ionic-liquid-multilayered graphene (MLG) interface.^40,41^ Therein, using a lab-based instrument, we reported on ion accumulation and the resulting electrical potential changes, only on top of the MLG electrode, which had a gold metal bottom electrode, and ionic liquid as the electrolyte. Hence, information derived was limited to only one electrode/electrolyte interface.

We now describe new work on two nearly equivalent multilayered-graphene electrodes deposited on a porous polyethylene membrane (PEM). As such, the system becomes a coplanar capacitor device, where one of the electrodes is electrified (source) and the other one is grounded (drain). This geometry allows us to investigate both interfaces as well as the electrolyte in between, as schematically shown in Figure 1a,b. Experimental details are given in the Supporting Information (SI).

**Figure 1:**
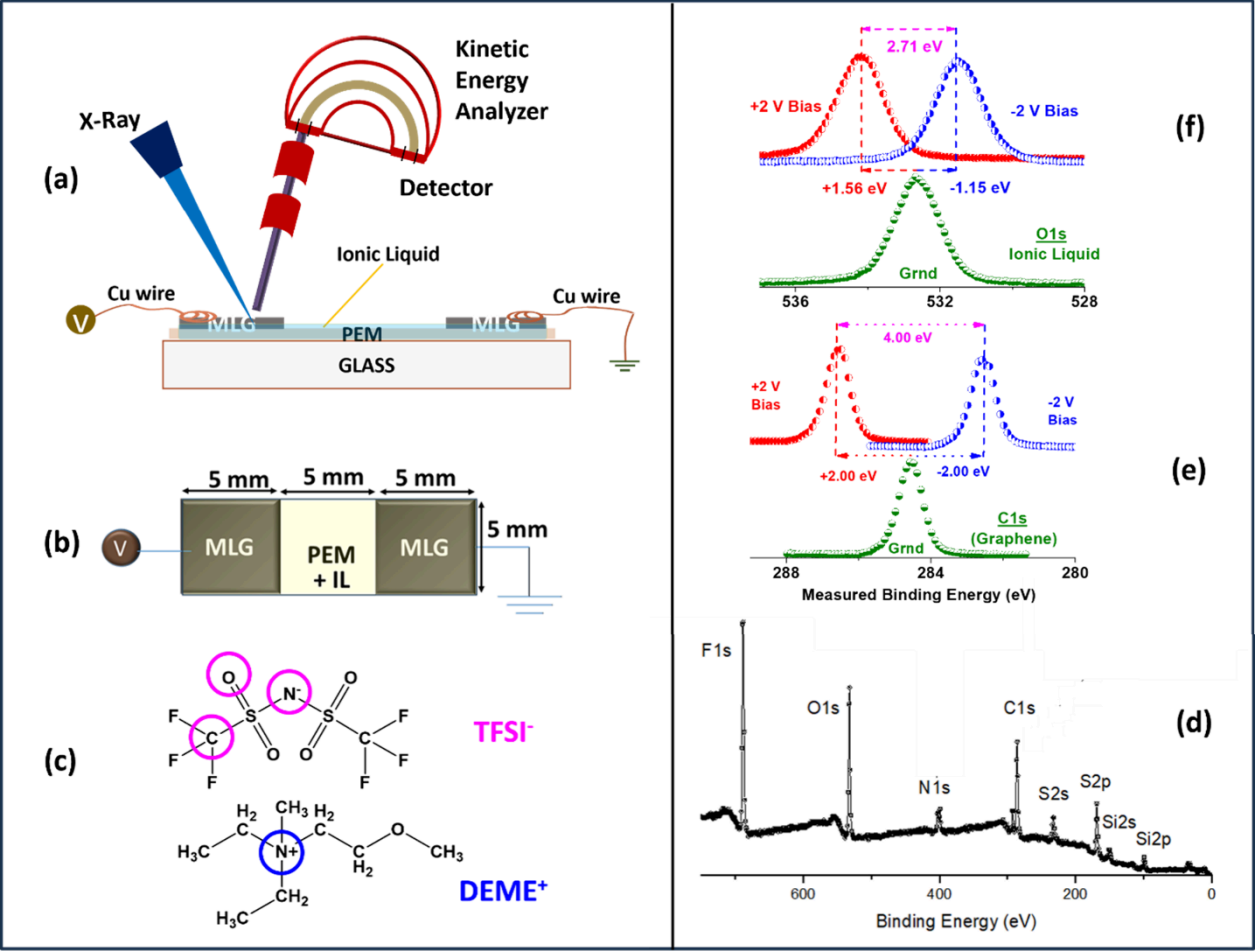
Schematic representation of XPS measurements on the co-planar capacitor device with one electrified and one grounded solid MLG electrodes, containing also the ionic liquid electrolyte; (a) side and (b) top views. (c) Chemical structure of the IL electrolyte (DEME-TFSI) and, (d) Its XP Survey Spectrum. (e) C 1s Spectrum of Graphene-Only without IL, recorded on the surface of the source MLG electrode, when it is Grounded and under +2 and −2 V DC bias. (f) O 1s spectra recorded on the same electrode after IL has been introduced, also when it is Grounded and under +2 and −2 V DC bias.

During XPS measurement, kinetic energies (K.E.) of the ejected photoelectrons are measured with a high precision, and using Einstein’s photoelectric effect formula (*hν* = B.E. + K.E.),^42^ binding energies (B.E.) of atomic levels of the ejected electrons are computed as also schematically shown in Figures S4 and S5 in the SI. In the formula, *hν* is the X-ray photon’s energy, which is the monochromatized Al Kα at 1486.6 eV in our instrument. The resulting spectrum consists of well-separated regions corresponding to the atomic core level peaks of the chemical moieties residing on the analyzed surface.^42^ A typical survey spectrum of the IL is shown in Figure 1d, and its chemical structure is given in Figure 1c. When constructing the device, the IL is introduced from the bottom of the PEM membrane, which spreads over and under the solid but porous carbon electrodes through wetting and/or creeping.^43^ Hence, their analyzed surfaces have a composite structure containing both solid graphene and liquid electrolyte phases. As a result, the C 1s region consists of features belonging to the electrode as well as to those of the electrolyte material, while the intense O 1s and F 1s peaks represent the anions of the IL electrolyte, as depicted in Figure S4b in the SI. As also indicated in the same figure, O 1s is inherently narrower than F 1s;^42^ therefore, it was chosen for recording potential variations of the IL medium throughout this work.

C 1s peak of graphene has a binding energy of 284.6 eV, when the device is grounded from both sides, which translates to (1486.6 – 284.6 = ) 1202.0 eV kinetic energy of the ejected photoelectron. If a DC Bias of +2.0 V is applied to the sample and XPS measurements are carried out on the electrified MLG electrode, the C 1s photoelectron’s kinetic energy is reduced to 1200.0 eV, since the applied positive bias decelerates the electron’s kinetic energy (red shift); as a result, the measured B.E. is now increased by exactly +2.00 eV, and −2 V bias causes the mirroring −2.00 eV blue shift, as both are depicted in Figure 1e. However, similar measurements on the O 1s peak representing the IL undergo less than the full 2.00 eV bias shifts, as shown in Figure 1 f. As we can gather, the IL medium under +2 V bias feels only +1.56 V electrical potential, reflecting the fact that the electrode’s potential is screened by the ions within the IL medium by −0.44 V. As such, recording the core level shifts under bias turns XPS into an independent chemical recording voltmeter. As we will also show below, extracting local electrical potential information on the source and the drain interfaces together provides a crucial and complementing link, not available through amperometric measurements.

As elaborated in Figure 1e,f, under bias and within the XP analyzed surface layer (400 μm diameter and ∼6–8 nm depth), the electrolyte peaks undergo different shifts in their positions due to screening of the electrode potential by the ions within the IL medium, leading to an electrical potential lower than the applied bias.^44–50^

Biasing also causes a noticeable increase in the apparent overall capacitance (*C* = *Q*/*V*) of the device, which is computed from the integrated current (accumulated charge = *Q*) after dividing by the voltage step (−0.5 to +0.5 V = ) of 1 V, as shown in Figure 2a and also in Figure S2 in the SI.

**Figure 2:**
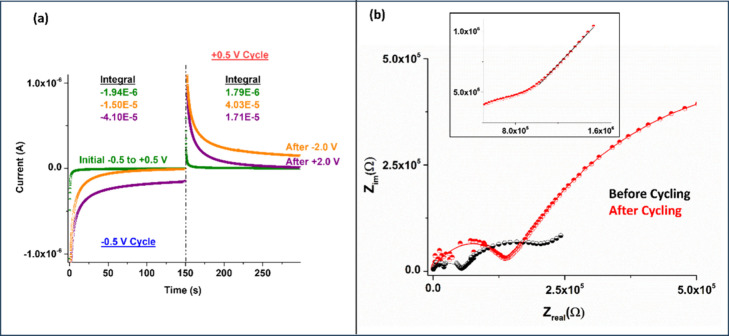
(a) Current measurements under −0.5 V and +0.5 V bias for a duration of 150 s each. The initial currents are shown with olive data points, which increase after application of −2.0 V bias for a duration of 30 min (1800 s) and becomes asymmetric (orange). Application of +2 V bias for the same duration also causes an increase in currents and creates asymmetry in the opposite direction (purple). (b) EIS measurements before and after ±2 V cycling for 14 h.

As is evident from the figure, capacitance increases more than 1 order of magnitude during the process, which can be attributed to being the result of electrosorption, leading to an increase in the apparent electroactive area of the system.^23^ Electrosorption effectively makes the surfaces more solvophilic, which, in turn, increases solvent wetting. This increased wetting increases the surface area and hence the capacitance.

Electrochemical impedance spectroscopy (EIS) measurements of the device before and after cycling are given in Figure 2b. Comparing the EIS data before and after cycling, the following information is derived:^51^ (i) The leakage resistances appear higher after cycling. (ii) Time constants are similar. (iii) A clear Warburg element is visible. The charges that are long-lived lead to higher resistance and the Warburg element, indicating that the diffusive component starts becoming more visible. This is reflected as a long-lived current in the time domain as well.^52^

Note also that a large voltage asymmetry can be created if only positive or only negative bias is applied for long periods, due to long-lived trapped charges,^23^ as exemplified in Figure S8 in the SI for another similar system. This property of the present electrochemical system forced us to employ long cycling with a 1 h period, using +2 V and −2 V bias for prolonged times of up to 14 h, before meaningful dynamical results, almost free of voltage asymmetry, could be obtained.

To capture the dynamics of charging, a relatively faster data acquisition procedure was employed, so that an entire regional spectrum (C 1s or O 1s) could be obtained with ∼0.5 s steps for 1 h, in the snapshot mode. Representative sets of spectra of the O 1s region are shown in Figure 3, during which the bias connecter is once opened and closed (open circuit), and at a later time, the connecter is once shorted (hard-grounded) and reconnected for a duration of ∼10 min each, while recording XP data continuously, both on the positive and negative cycles, as well as on both the source and the drain electrodes. In Figure 3a,b, only the data recorded on the source electrode are shown, each of which consists of ∼8000 O 1s spectra.

**Figure 3:**
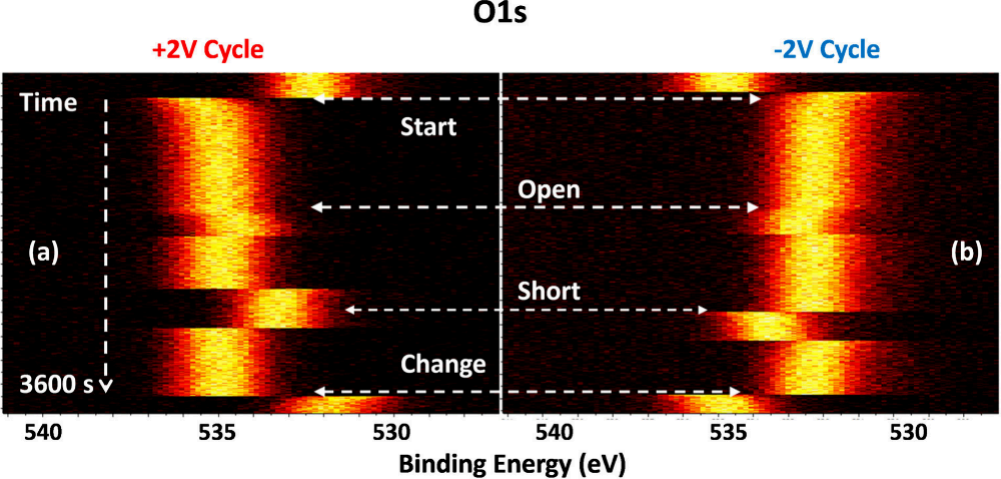
Heat-scale representation of **∼**8000 XP spectra of the O 1s region, recorded iteratively in the snapshot mode within a ∼0.5 s window, while under cycling: (a) +2 and (b) −2 V bias for 1 h on the source electrode. During each cycle, the circuit was once opened and once shorted for ∼10 min.

All of the O 1s spectra are individually fitted, and their binding energy (B.E.) positions have been obtained to extract the temporal variations in the local electrical potential on both electrodes, which are displayed in Figure 4.

**Figure 4:**
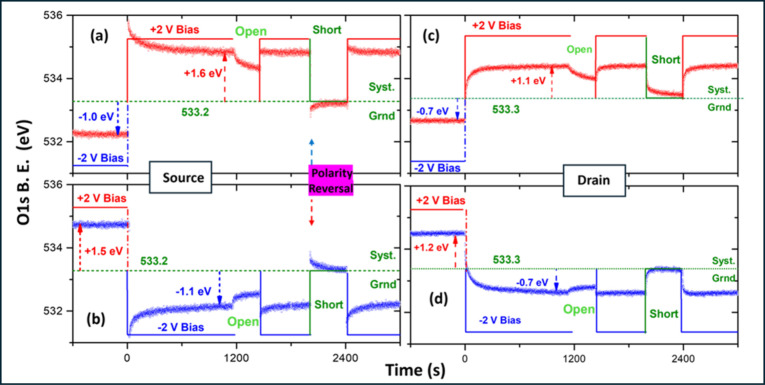
O 1s B.E. variations extracted from the data recorded on the source (a and b) and the drain MLG electrodes (c and d), for 1 h under +2 and −2 V bias, respectively. Within this period, the circuit was once opened and once shorted for a duration of ∼10 min, while XPS data were recorded continuously.

When the sample is grounded and before biasing, the O 1s peak, representative of the IL medium, has a B.E. position of 532.6 eV (not shown), when and if the IL medium carries no charges at all. However, as we will demonstrate shortly, the device always carries some charge, depending on its electrochemical history.^21^ The resulting electrical potential of the IL at the source electrode can be determined as (533.2 – 532.6 = ) +0.6 V from the O 1s B.E. position of the system’s ground, also indicated in Figure 4a,b. We extract the system’s ground position when the connector is shorted and the system decays asymptotically to its equilibrium state from both biases.

It is also important to note that we start recording XPS data for the positive cycle ∼15 min before the beginning, where the IL medium is at the end of its negative cycle and is already positively charged, as evidenced by the B.E. position of the O 1s at 533.2 eV in Figure 4a, at time equals zero position. This number indicates that the applied −2 V bias is screened by +1.0 V by the cations of the IL medium, which is now carrying +1.0 V of ionic charge, computed from the measured B.E. (−2.0 + 1.0 = −1.0 V) at the start of the +2 V cycle. Therefore, upon imposing +2 V, the O 1s peak jumps up to 533.2 + 1.0 + 2.0 = 536.2 eV and decays slowly down to ∼534.8 eV (= 533.2 + 1.6) toward the end of the cycle but stays always positively charged. When the circuit is opened (OCP), it starts decaying slowly down but again stays above the grounded position. However, a polarity reversal is observed when the electrode is shorted, such that its position jumps down to the negative region and starts decaying up toward the system’s ground value. A mirroring polarity reversal is captured during the −2 V cycle, as shown in Figure 4b. These surprising changes manifest as enhanced changes but without polarity reversal on the drain electrode, as depicted in Figure 4c,d.

Parallel measurements on the C 1s region for following the voltage developments on the graphene electrodes are more difficult because of the overlapping features, as was displayed in Figures 1 and 2, and require higher fidelity data for employing curve-resolving procedures. Therefore, we have recorded the C 1s and O 1s regions on top of both electrodes, also iteratively, using the slower (1.5 min each) but more accurate scanning mode. The results are given in Figure 5, as XP data recorded while cycling +2 and −2 V biasing every 30 min, during which the circuit was also once opened and once shorted.

**Figure 5:**
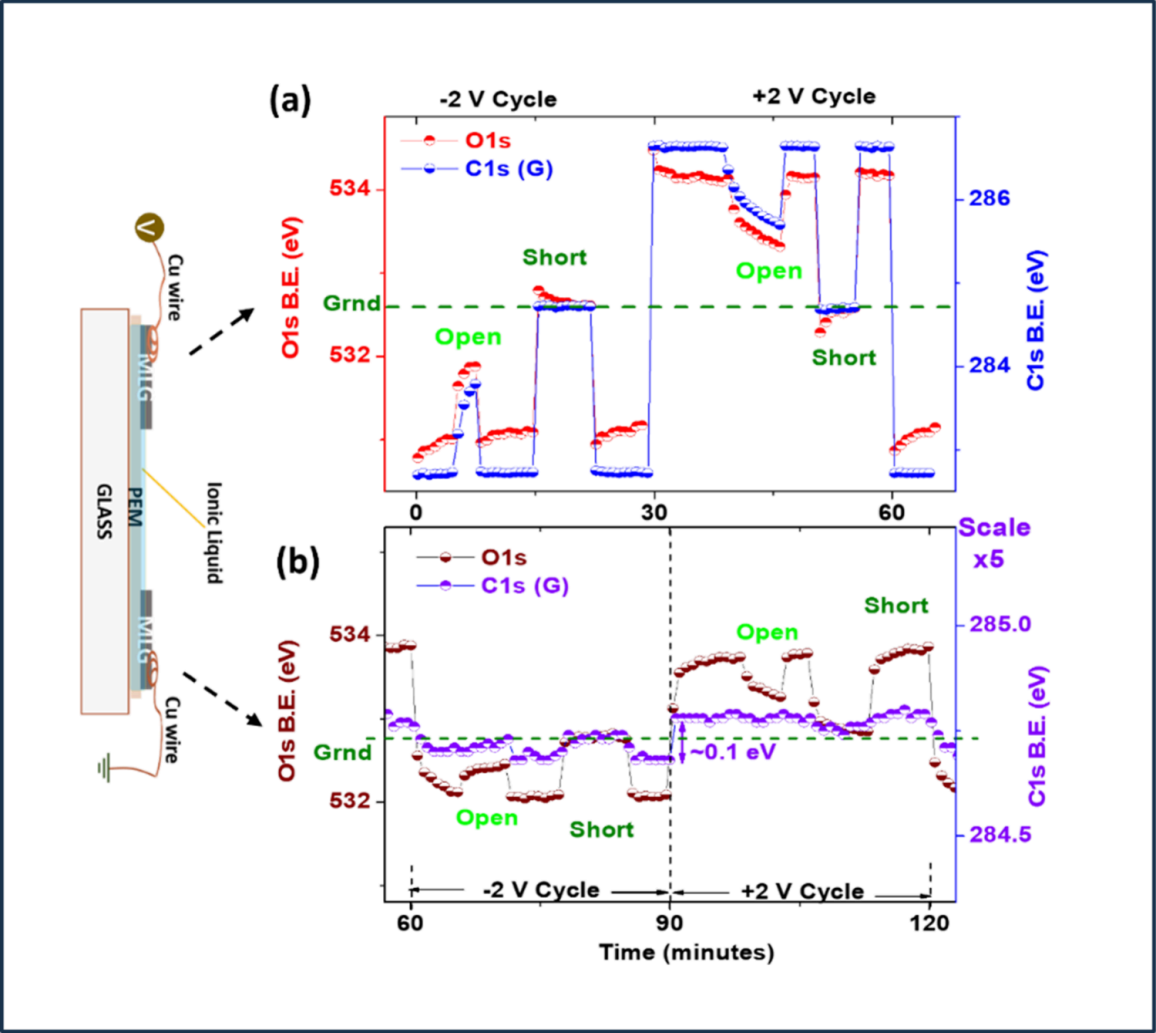
O 1s B.E. variations recorded on (a) source and (b) drain MLG electrodes, for 1 h under +2 and −2 V bias. Similarly, within this period, the circuit was once opened and once shorted.

The extracted B.E. positions of the C 1s representing the MLG electrode, and the O 1s representing the IL medium, are reproduced in Figures 5 a. The surprising opposite shifts in the O 1s position are mirrored, when the sample was temporarily shorted. Moreover, a small (∼ +0.1 eV), but nevertheless measurable shift in the graphene C 1s position is also captured, which is shown in Figure 5b in the 5 times expanded B.E. scale. This finding indicates that biasing also induces small but finite charge-trapping sites with comparable time-constants of the IL medium within the graphene layers,^41^ related most probably to the defects created during electrosorption and/or other processes.^26^

These unexpected new findings can be comprehended if one considers the dynamics of the EDL formation in porous carbon-based electrodes, since multifaceted nonplanar and interconnected surfaces and their interfaces are involved, as opposed to the classical description of one planar metallic electrode–liquid electrolyte interface.^12,28–30^ Our understanding of the mechanism of the observed polarity reversal, when the device is shorted, is schematically summarized in Figure 6, where part of the data displayed, corresponding to the shorting part of Figure 4, is reproduced in more detail.

**Figure 6:**
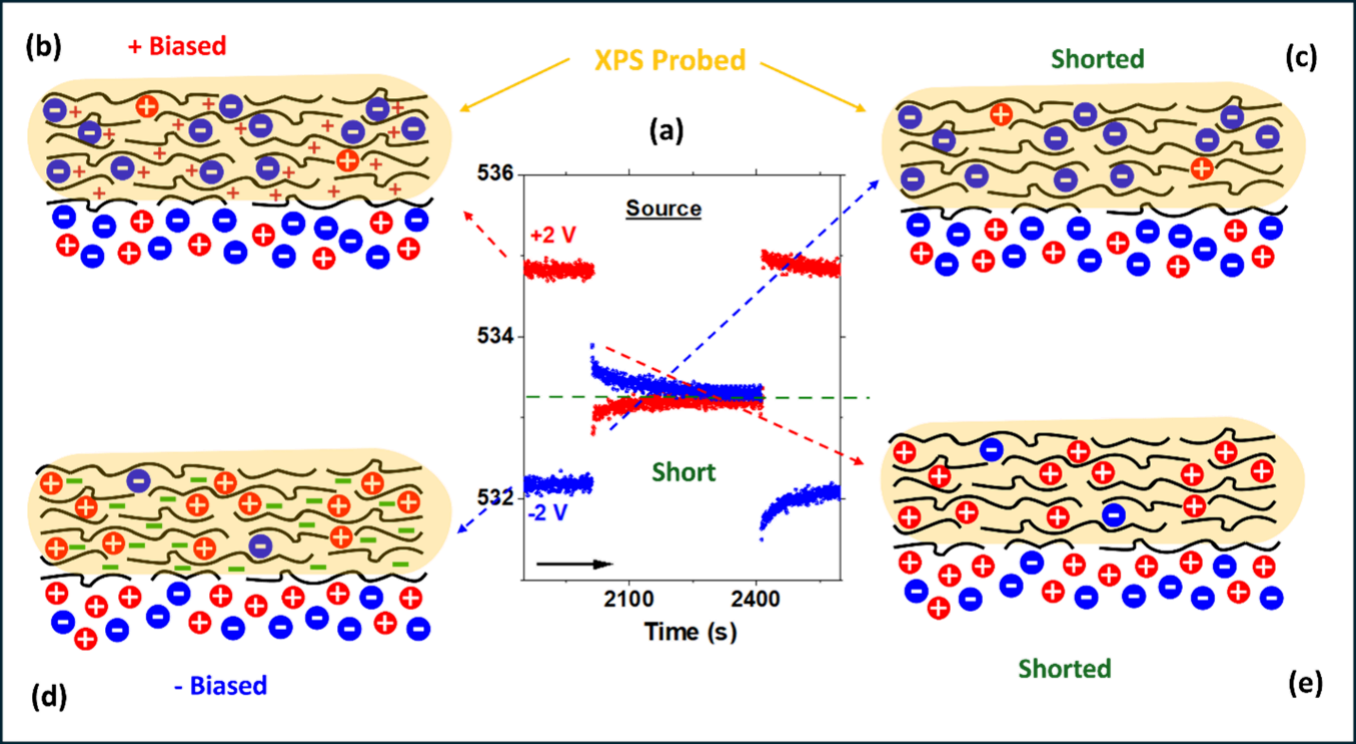
(a) Parts of the O 1s B.E. variations recorded on source MLG electrode, for 1 h under +2 and −2 V bias, just before, during, and after shorting periods, which are the same as displayed in Figure 4. The cartoons display schematically the electronic and ionic charges, under +2 V bias (b and c) and −2 V bias (d and e), on the MLD and ionic IL domains before and after shorting the bias connector.

As schematically summarized in the cartoons of Figure 6, upon +2 V biasing the source electrode is immediately charged positively, by creating holes within the graphene matrix, and anions of the IL nearby move to screen it with much slower time-constant(s); hence, the overall potential experienced by the IL medium decays down toward the equilibrium potential, which is about +1.6 V, as was also indicated in Figure 4a. When shorted, the holes on the electrode are filled immediately by the electrons from the ground, and the IL is left in its negative but only ionically charged state, which is approximately −0.4 V, and starts decaying toward the shorted value of 0 V. The process is complemented by the opposite changes on the drain side, which is initially grounded but starts slowly acquiring negative charges with respect to the source electrode, upon +2 V biasing, and therein the cations nearby start slowly screening it. Hence, when grounded, the IL is left with extra cations, i.e., positively charged, and starts decaying down toward 0 V, the equilibrium state. The polarity reversal is mirrored under −2 V bias, as depicted in Figure 4b.

In our previous work, although we had observed and reported dynamical polarity reversals between the electrified and grounded electrodes, polarity reversal on the very same electrode upon shorting has never been observed, nor reported by us or by others before.^41–51^ The reason why we are able to observe it now must be related with creating heavily electrosorbed and nearly equivalent multilayered graphene electrodes realized in the present work.

A significant research effort has been devoted to better understanding, at the molecular level, the EDL structure, capacitance, and charging dynamics, using both theoretical and experimental methods, and the subject was recently reviewed by J. Wu.^26^ As was also highlighted in that work, Janssen and co-workers have reported on capturing the slow charging dynamics of EDL formation within carbonaceous supercapacitors, using coulometry and calorimetry, and also on development of a successful theoretical framework using the transmission line model.^28–30^ For a similar device, their observed and calculated time constants were reported to be 10^2^–10^3^ s, which match our XPS-derived two time constants of 20 and 200 s as shown in Figure S7 of the SI.

In summary, we show, using o-XPS, that dynamics of the graphene/ionic liquid interfaces can be followed in ultimate detail by distinguishing the contributions of electronic and ionic components of the local electrical potentials with complementary current measurements. The results presented here clearly exhibit the difference in time scales for the charging dynamics of the electrodes and the electrolyte. However, further detailed experimental and theoretical works are needed to elucidate the roles and functions of the various components of electrochemical systems. The methodology introduced is very simple and can be adopted by any lab- or synchrotron-based XPS instruments. We plan to carry out experiments along these lines and hope that others will join us in furthering and elevating our understanding of the two-century-old EDL phenomenon.

## Supplementary Material



## Notes

Details of materials and methods used, emphasizing especially electrical biasing of the devices used in this work, together with additional data for electrical current and local electrical potential variations (PDF)
