# Supplementary material for: Unveiling Ionic/Electronic Contributions to the Potential Development of Electrical Double Layer Using XPS
Source: J Phys Chem Lett. 2025 Aug 20;16(34):8778–84. doi: 10.1021/acs.jpclett.5c01855 (PMC12400400; doi:10.1021/acs.jpclett.5c01855)
Supplement: Supplementary file 1 [file jz5c01855_si_001.pdf]

Supporting Information

Unveiling Ionic/Electronic Contributions to the Potential Developments of Electrical Double Layer using XPS

Ezgi Kutbay,[a] Burak Ulgut,[a] Coskun Kocabas[b] and Sefik Suzer[a],*

[a]Department of Chemistry, Bilkent University, 06800 Ankara, Turkey
[b]Materials Department, Manchester University, Manchester M13 9PL, United Kingdom
*Corresponding author (suzer@fen.bilkent.edu.tr)

Experimental Details

Multilayer graphene samples are grown on nickel foils (Alfa Aesar) using chemical vapor deposition at temperatures ranging from 850 to 1000 °C at ambient pressure. A mixture of H_2, Ar and CH_4 gases is used during the growth with flow rates set as 100 sccm, 100 sccm, and 30 sccm respectively, and the growth time is 5 mins. This procedure enables fabricating self-standing multilayer graphene films having 300 to 600 layers. Two 5x5 mm MLG film electrodes, separated by 5 mm, are transferred onto the Porous Polyethylene Membrane (PEM), which is placed on a glass slide and the ionic liquid is introduced below the membrane, which eventually permeates and covers the electrodes. The ionic liquid, [*N*,*N*-Diethyl-*N*-methyl-*N*-(2-methoxyethyl) ammonium bis (trifluoromethanesulfonyl) imide] DEME-TFSI, is purchased from Io-Li-Tec and used for fabricating the co-planar devices. A schematic representation of the device, together with an SEM image of the MLG are given in Figure S1.

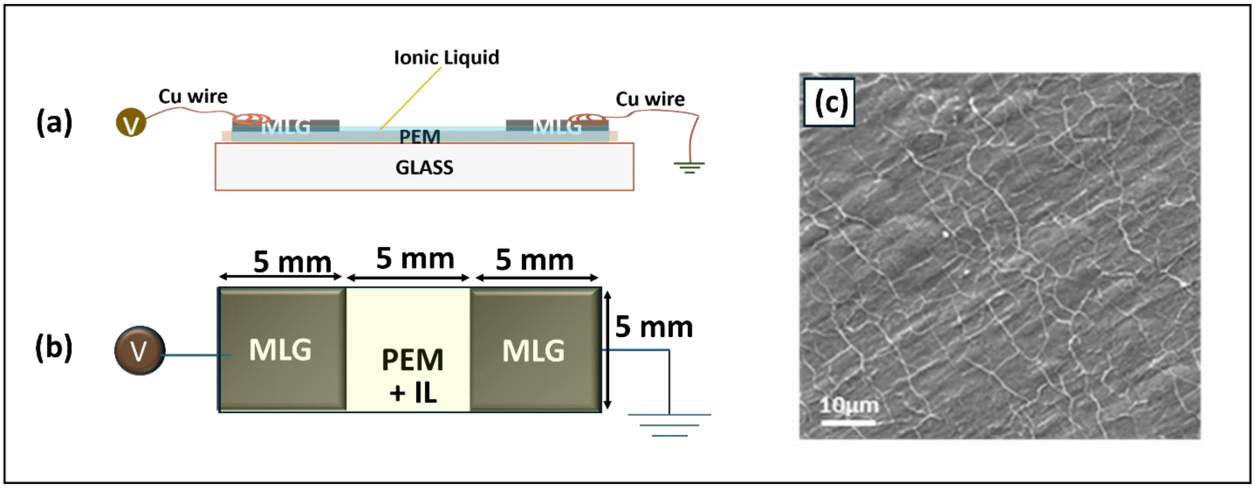

Figure S1. The Co-Planar Capacitor Device; **(a)** Side and **(b)** Top Views. **(c)** Secondary Electron Microscope Image of the pristine MLG surface.

A Thermo-Fisher K-Alpha X-ray Photoelectron Spectrometer with a monochromatized photon energy of 1486.6 eV has been used to collect data for all measurements. External bias is applied to one of the MLG electrodes using a Keithley 2400 Source-meter, and the other electrode is grounded. After introducing the device into the UHV system of the spectrometer, the system including the device is heated to 80 ^oC overnight to remove residual water and other volatile impurities. XP Spectra, both with and without external bias, as well as the induced current, have been recorded simultaneously. XPS data processing is carried out using the Avantage software package provided by the manufacturer.

Measurements

a- Simple I-V Measurements

Due to the large capacitance of the device, induced currents under bias have strong dependence on devices' history, the polarity and the extent of the bias applied. This feature is shown in Figure S2 below. The apparent capacitance (C = Q/V) of the device, which is computed from the integrated current (accumulated charge = Q) after dividing by the voltage step (-0.5 to +0.5 V =) of 1 V, as shown in Figure S2 (a), and also in Figure 2 (a) in the main text. Accordingly, the capacitance of the system increases more than an order of magnitude during various biasing processes, which can be attributed to being the result of electrosorption, leading to an increase in the apparent electroactive area of the system.[1]

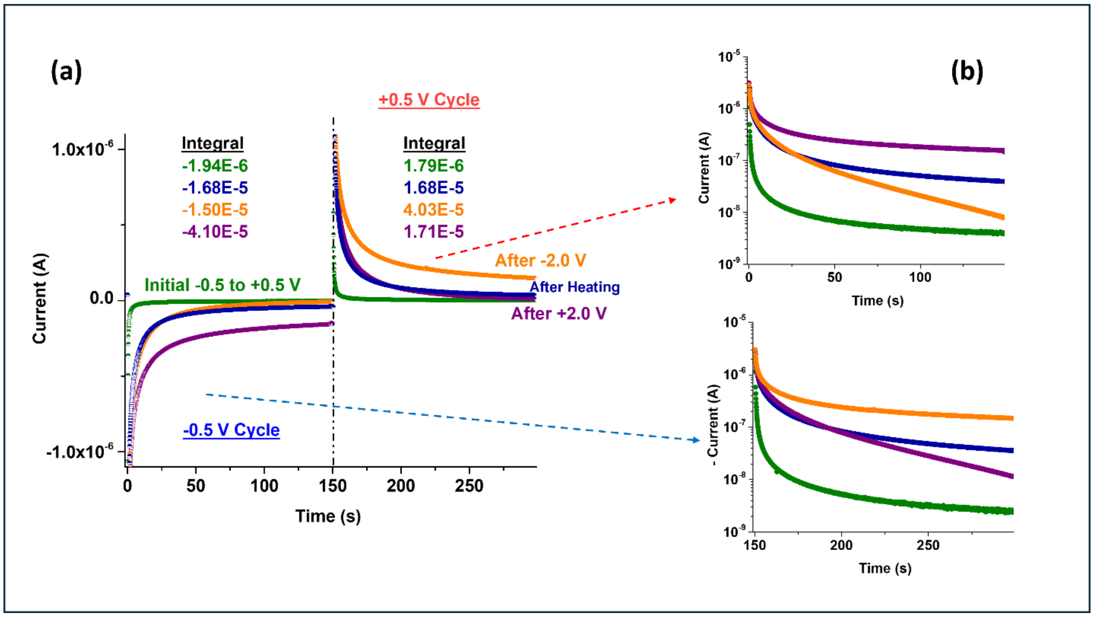

Figure S2 (a). Current measurements under **-0.5 V** and **+0.5 V** bias for a duration of 150 s each. The initial currents are shown with **olive** data points. After application of **-2.0 V bias** for a duration of 30 minutes (1800 s) current increases even more and becomes even more asymmetric (**purple**) in the opposite direction**. (b)** The same data plotted in a semi-logarithmic fashion.

As can be gathered from the figure, initial values are very low, but after application of negative 2 V bias for a duration of 30 minutes (1800 s), the current increases for more than 1 order of magnitude and becomes strongly asymmetric. Conversely, upon the application of positive 2 V bias for the same duration, the current increases even more and becomes even more asymmetric in the opposite direction. As also discussed in the main text, these findings point out the process of electrosorption, which leads to an increase in the apparent electroactive area of the device.[1] Therefore, to obtain meaningful measurements, we had subjected the devices to very long -2 and +2 V biasing for a duration of 1 hour each-way for a total of 14 hours (7 cycles).

Estimation of the overall capacitance value of the device using the current and Electro-Chemical Impedance Spectroscopy (EIS) measurements gave a value of ~250 µF. Gravimetric capacitance of our device can also be estimated as ~3 F/g, using the weight of the 2 MLG electrodes, which was ~80 µg. This capacitance is naturally much lower compared with those of the state-of-the-art carbon capacitors, exceeding > 100 F/g.[2]

b- Electrochemical Impedance Measurements

Comparing the EIS data before and after cycling, the following information is derived.[3] (i) The leakage resistances appear higher after cycling, (ii)Time constants are similar (iii) A clear Warburg element is visible. The charges that are long lived lead to higher resistance and the Warburg element, indicating that the diffusive component starts becoming more visible. This is reflected as a long-living current in the time domain as well.[4]

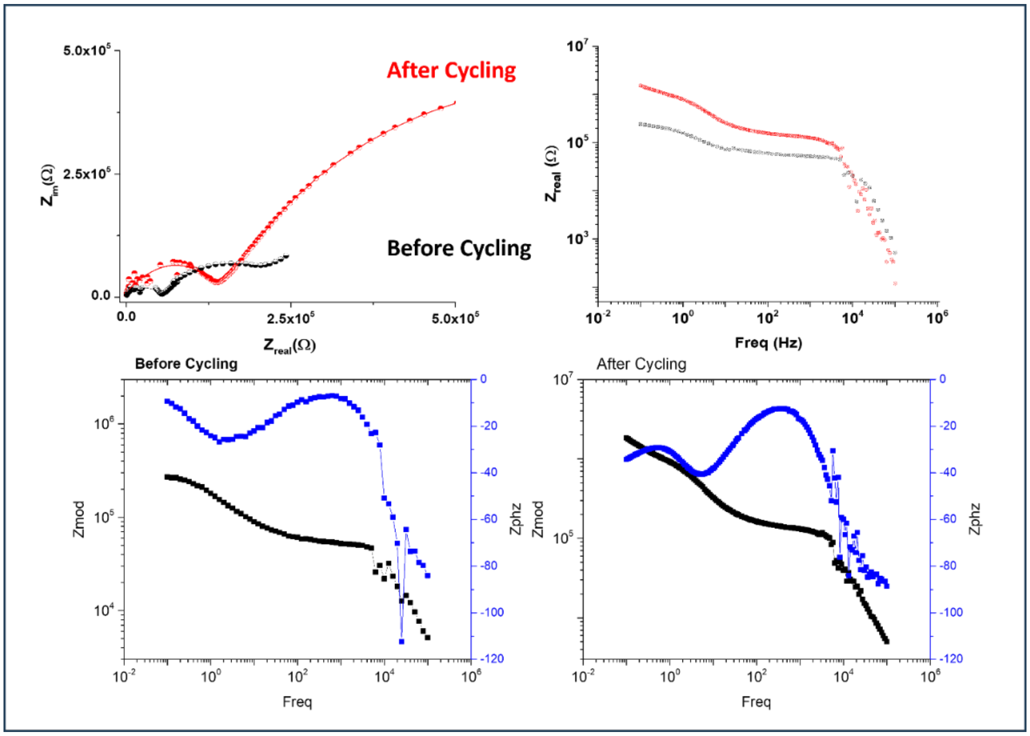

Figure S3. EIS Measurements before and after Cycling for 14 hours.

c- XPS Measurements

A typical XP Survey Spectrum of the IL is shown in Figure S4 (a), where all peaks of the IL are visible, together with Si2s and 2p impurity peaks. XPS is a strong chemical analysis tool, hence identity of all constituent atoms of the IL, except for hydrogen, are represented as corresponding peaks at certain binding energies, well separated in the energy scale.[5] Moreover, chemical signature of the atoms are also resolved by the corresponding shifts (chemical shift) in that scale. Accordingly, whereas only one fluorine peak bonded to carbon ($-CF_3$), one oxygen peak bonded to sulfur (SO_2) and one sulfur peak is observable, two separated nitrogen peaks are observable corresponding to the anionic and cationic moieties, as shown in Figure S4 (b). In the C1s region, there are several overlapping peaks representative of both ions, and another well separated peak where the carbon is bonded to the strongly electronegative F atom ($-CF_3$). The technique is also quantitative, where the area under each peak, corrected for the photoelectron cross-section can be used to extract relative atomic composition. For example, the second peak in the C1s region represents twice as many carbon atoms, compared to the first and third peaks. For the co-planar capacitor device investigated in this work, having two multilayered-graphene electrodes and IL electrolyte, the C1s region becomes even more congested with the additional C1s peak of the

graphene, as shown in Figure S4 (e), which can nevertheless be faithfully deconvoluted using a curve fitting procedure.

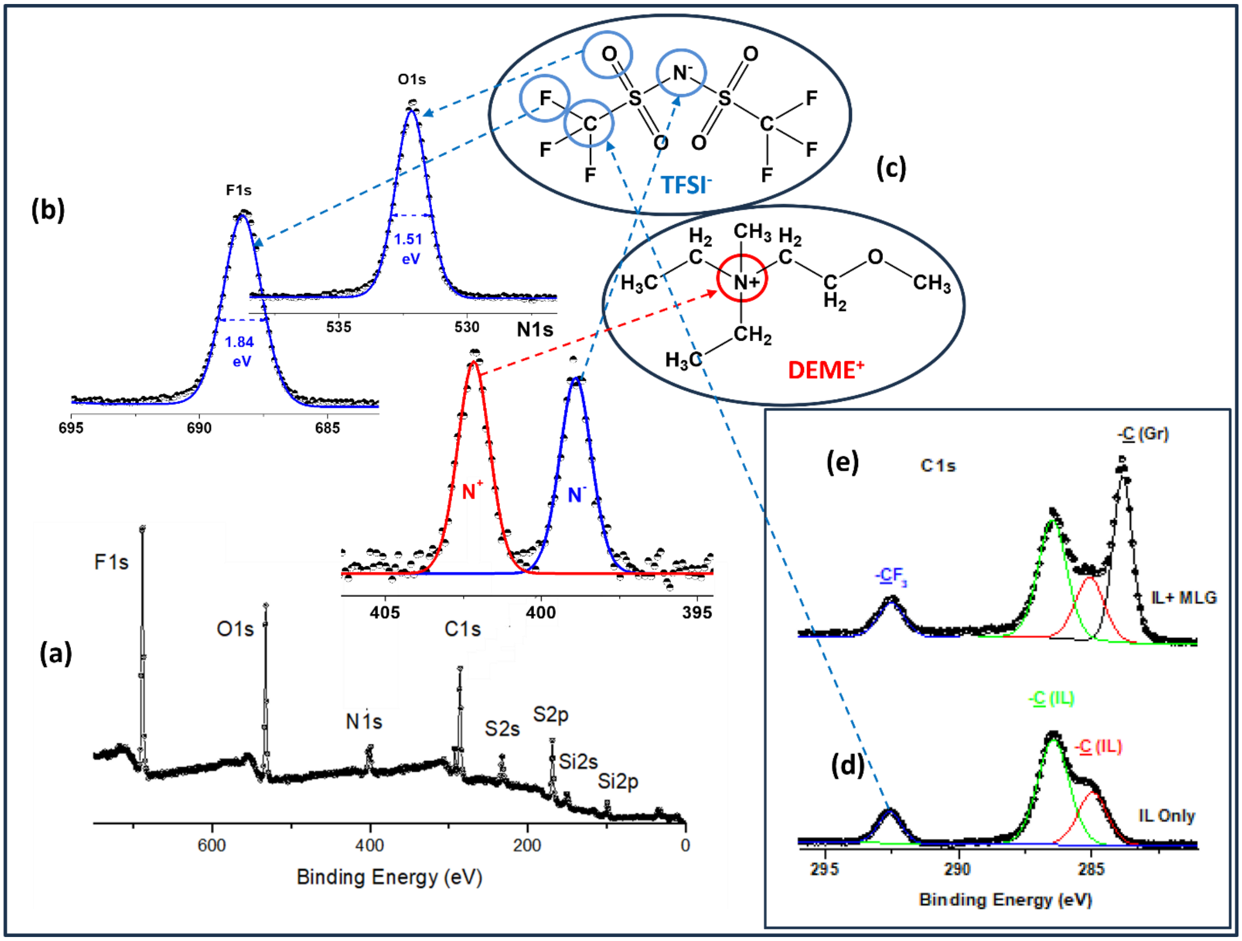

Figure S4. **(a)** Survey XP Spectrum of the IL. **(b)** Detailed scanned spectra of F1s, O1s and N1s regions. **(d)** C1s region of the IL only, and **(e)** on top of the Multilayered Graphene Electrode, Chemical Structure of the IL, with its anionic and cationic species are shown in **(c)**.

On the other hand, the main trust of the present work utilizes another strong but underused capability of the technique, which is related to the possibility of directly reflecting the local electrical potential of the medium where the corresponding atoms reside. XPS measures the kinetic energy (K.E.) of the ejected photoelectrons with high precision and using the well-known Einstein's photoelectric effect formula (**hν = B.E. + K.E.**), gives the binding energy (B.E.) of the atomic level of the ejected electron as schematically shown in Figure S5. In the formula, **hν** is the X-Ray photon's energy, which is the monochromatized **AlKα** at 1486.6 eV, in our instrument.

The technique is also surface sensitive, since the created electrons carry electrical charge, they are severely scattered by the condensed medium (solid and/or liquid) of the sample, hence only the

electrons created within the top 0-8 nm survive with their unaltered kinetic energies. Furthermore, to prevent additional scattering by the gaseous medium, all measurements are performed under ultra-high vacuum conditions.[5]

d- XPS Measurements Under Bias

Under standard analyses conditions, the sample is connected to the spectrometer's ground, as a result the Femi level of the solid/liquid sample is equilibrated with that of the spectrometer. When the binding energies are calculated they are also corrected with respect to the work function of the spectrometer, as also shown in Figure S5 (a). For the Multi-Layered-Graphene used in this study, the C1s level's binding energy is equal to 284.6 eV, when the device is grounded from both sides, which translates to (1486.6 – 284.6 =) 1202.0 eV kinetic energy of the ejected photoelectron.

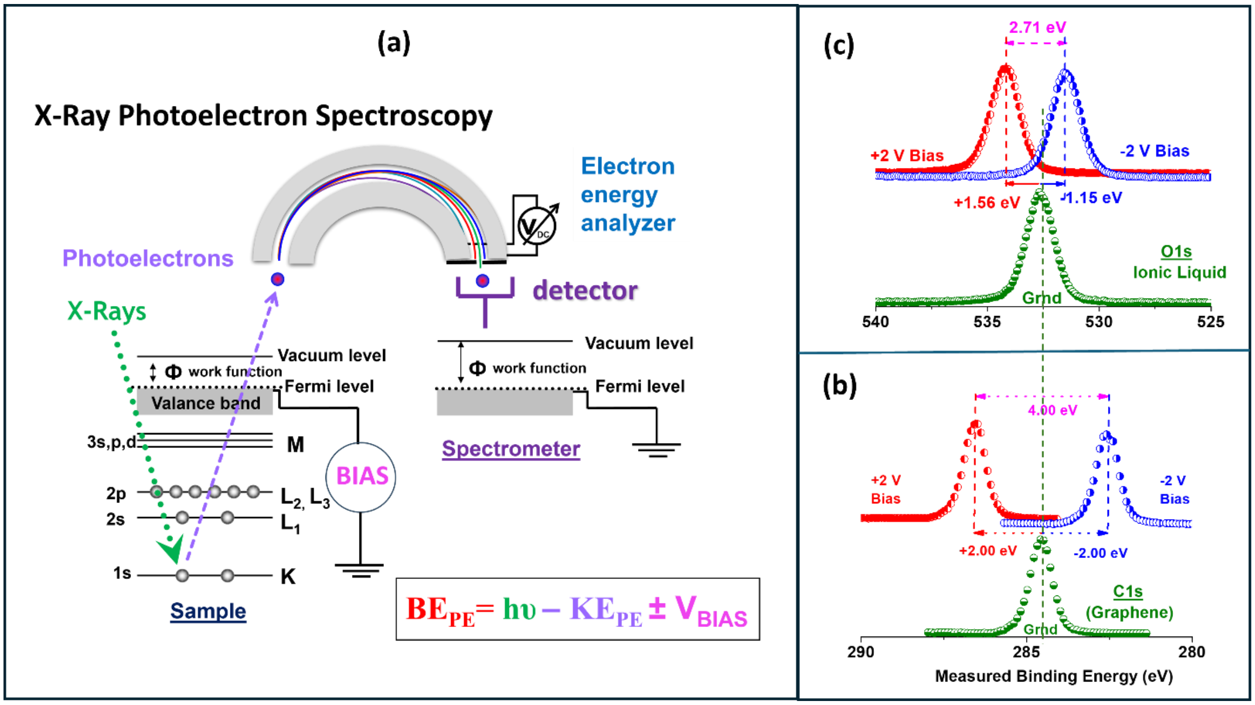

Figure S5 (a). Schematics of the XPS measurements. **(b)** C1s region of the pristine Multi-Layered Graphene Electrode and **(c)** O1s region of the Ionic Liquid, recorded; when grounded (**green**) and under **+2 V** and **-2 V** bias, respectively.

If we apply a DC Bias of +2 V to the sample, the C1s photoelectron's energy is reduced to 1200.0 eV, since positive potential impedes the electron's kinetic energy (red shift), as a result, the measured B.E. is now increased by exactly +2.00 eV as shown in Figure S5 (b). Negative (-2 V)

Bias causes the mirroring -2.00 eV blue shift, yielding a total of 4.00 eV between the two biased conditions. These shifts are referred to as trivial shifts and are used only to ensure the validity of the method.[6] However, similar measurements on the O1s peak representing the IL undergoes less than the full 2.00 eV bias shifts, as shown in Figure S5 (c). As we can gather, the IL medium under +2 V bias feels only +1.56 V electrical potential, reflecting the fact that the electrode's potential is screened by the ions within the IL medium, which is equal to +0.44 V. Under -2 V bias, the screening is larger -0.85 V, giving a total difference of 2.71 eV and signaling the presence of an effective screening of (4.00 -2.71=) 1.29 eV. **Note that this is the most direct and relatively non-invasive method of measuring the local electrical voltage developments, via the chemical voltmeter, not available by any other technique.**

Such chemically specific local voltage developments can also be measured/captured in a time-dependent fashion, which has been outlined in the main text in Figures 3 and 4, and displayed in Figure S6.

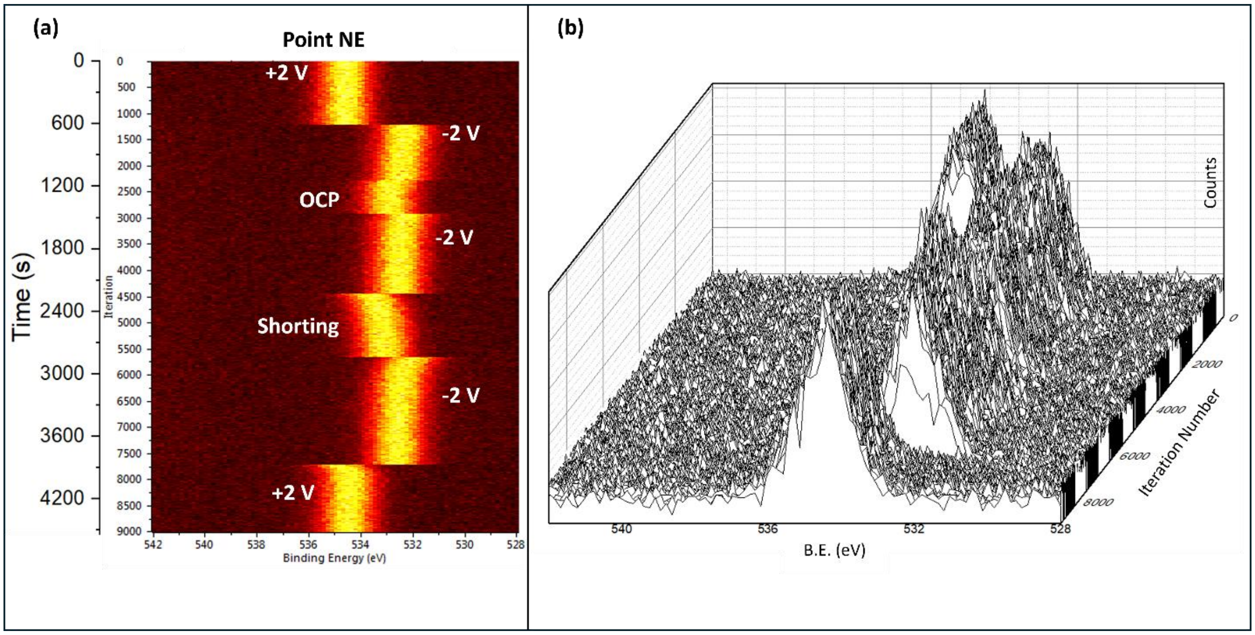

Figure S6 (a). Two-dimensional (2D) time-resolved color map showing the evolution of O1s core-level spectra over 9000 consecutive acquisitions with temporal resolution of 0.5 seconds collected on the electrified MLG Electrode for one hour. During data collection, the connecting cable was once opened for ca. 10 minutes and closed and once shorted for also ca. 10 minutes and closed. **(b)** Three-dimensional (3D) plot of the same dataset showing how the intensity and the position of the peak shift over the 9000 iterations.

The voltage variations, as reflected by the binding energy variations of the O1s peak of the XPS data, and simultaneous current measurements under positive and negative cycles, both recorded at the electrified electrode, are displayed in Figure S7. Similar measurements for only the voltage

variations at the grounded electrode are given in Figures 4 (a) and (b) in the main text. Although the current measurements have also been recorded at the grounded (drain) electrode, they are not reproduced here, since they are identical to those shown in Figures S7.

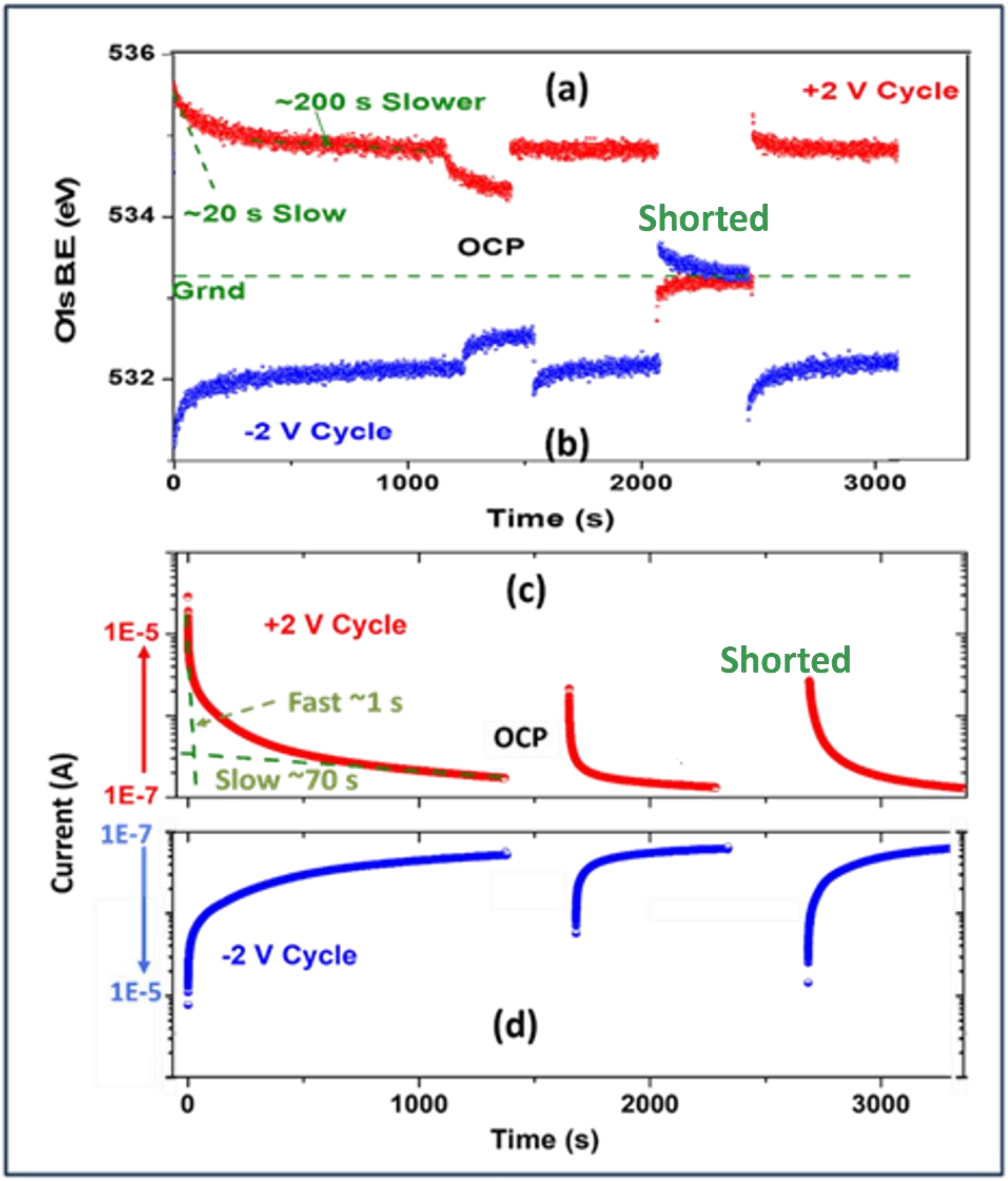

Figure S7. Variations in the XPS O1s B. E. position **(a)** and **(b)**. Current measurements, simultaneously recorded under; **(a)** +2 V and **(b)** -2 V bias, respectively, for a duration of one hour (3600 s) each. Note that the changes in the B.E. position are much smaller and slower, when compared to the current variations, hence the latter are displayed on semilogarithmic scales in opposite directions. Only the measurements on the electrified electrode are displayed.

The figure also emphasizes the presence of a multitude of time constants and that the XPS derived voltage variations are slower, when compared with the current variations.

As was also mentioned within the main text, very asymmetric voltage variations can also be recorded, depending strongly on the history of the device, in terms of various biasing conditions, as exemplified in Figure S8, where even under -2 V Bias, the measured electrical potential stays almost always in the positive region, revealing the fact that both the source and the drain electrodes carry remnant charges, charge-memory effect.[7]

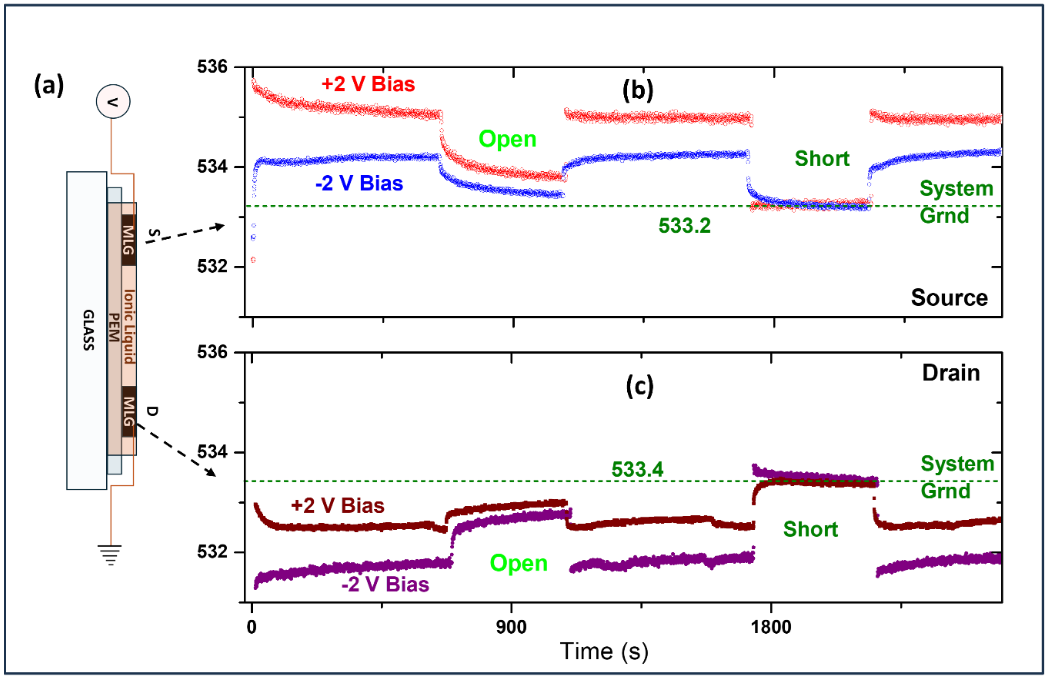

Figure S8 (a). An Extremely Asymmetric Device. Variations in XPS O1s B. E. position under +2 V and -2 V Bias, for a duration of 1 hour (3600 s) each on; **(b)** The Source and **(c)** The Drain Electrodes.

e- Voltage Dependence of the Time Constants

We also checked dependence on the amplitude of the bias and recorded similar data by varying sequentially the bias from 0.5 to 2.0 V as displayed in Figure S9. Examination of the figure even after plotting it in a semi-logarithmic fashion reveals that the time constants do not have a strong dependence on the amplitude of the bias voltage.

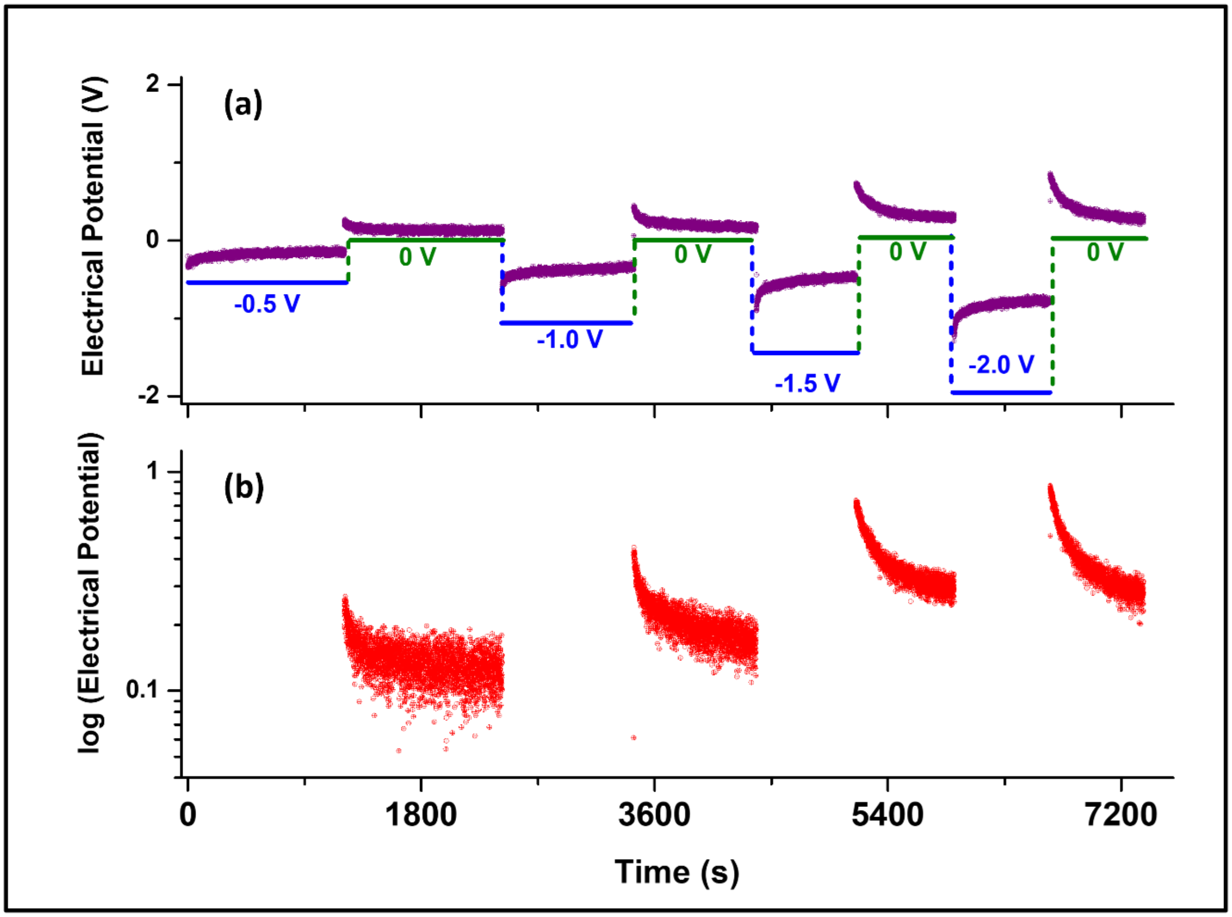

Figure S9. Voltage dependence of the electrical potential variations, extracted from the O1s B.E. positions**. (a)** Under only **Negative -0.5 V** to **-2.0** V excitations, each for a duration of 900 s. After each bias the device is subjected to **0 V**, i.e. **Shorting**, for also 900 s long. **(b)** Semi-logarithmic plot of the same data showing only the positive voltages after shorting.

f- Chemical and Electrical Quantification of the MLG Electrode before and After Cycling

Another important property of XPS is its ability to provide quantification, for which the measured chemically specific atomic % compositions are given in Table S1 below.

		Source			Drain	
**Species**	**B.E. (eV)**	**At. % Before**	**At. % After**	**B.E. (eV)**	**At. % Before**	**At. % After**
**C1s (Graphene)**	284.6	31.7	21.9	284.6	40.2	35.2
**C1s - CF_3 (IL)**	292.8	3.5	4.7	292.8	3.5	3.2
**F1s (IL)**	688.8	14.3	19.3	688.7	12.9	14.2
**$N1s^-$ (IL)**	399.4	2.1	-	399.3	1.8	-
**$N1s^+$ (IL)**	402.7	2.2		402.7	1.7	

If we consider the composition on the section probed by the X-Rays on the source electrode, its volume can be estimated as follows:

$$\textbf{Volume (probed) = Area x Depth} = [400 \times 200 \times 10^{-12}\ m^2] \times (6 \times 10^{-9}\ m) = 5 \times 10^{-16}\ m^3$$

From the table we gather that there are approximately 10 graphene carbon atoms for every IL molecule, which has **2 C atoms (2 x CF_3 groups)**, therefore the atomic carbon ratio is ~ **20:1 (Graphene/IL)**. As a result, we can safely assume that MLG matrix has not been perturbed significantly both before and after the electrosorption process, hence the density of graphite, which is **2 g/cm^3**, can be used to estimate that **5 x 10^{13}** C **atoms and 2.5 x 10^{12} IL molecules** have been probed.

One further step can be taken to estimate the number of IL ions electrosorbed to provide the screened voltages we observe (in the order of about 1 V), we can use the following formula.[7]

$$Q = \Phi \times (4\pi\varepsilon\varepsilon_0(A/d))$$

For this formula, we need the electrochemically active area. As a rough estimate, 40 μC of charge (integral from Figure S2) yields 40 μF of capacitance given the applied 1V. As indicated by Bard et.al[4] as well as others, 1cm^2 of electroactive area yields anywhere from 10-40 μF or double layer capacitance. For this system, we will use 1 cm^2 for this estimation.

$$Q = 1\ V \times [4\pi \times 20 \times 8.85 \times 10^{-12}\ (F/m) \times (10^{-4}\ m^2/10^{-9}\ m)] = 2 \times 10^9\ C$$

Where **ε** is the dielectric constant of the IL (~20),[9] and **d** is the estimated thickness of the Debye Layer, taken as 1 nm.[4] This simple estimation gives us the total ionic charge needed to be accumulated to yield a measurable electrical potential of 1V to be unphysically large. This indicates that a simple Coulombic model with the bulk dielectric constant fails to explain the electrochemical double layer.

References

1- Liu, X.; Lyu, D.; Merlet, C.; Leesmith, M. J. A.; Hua, X.; Xu, Z.; Grey, C. P.; Forse, A. C. Structural Disorder Determines Capacitance in Nanoporous Carbons. *Science (1979)* **2024**, *384* (6693), 321–325. https://doi.org/10.1126/science.adn6242.

2- Wu, J. Understanding the Electric Double-Layer Structure, Capacitance, and Charging Dynamics. *Chem Rev* **2022**, *122* (12), 10821–10859. https://doi.org/10.1021/acs.chemrev.2c00097.

3- Orazem and Tribollet; *Electrochemical Impedance Spectroscopy*, *2nd Edition*, John Wiley and Sons, NY, USA **2017.**

4- Bard, Faulkner and White, *Electrochemical Methods: Fundamentals and Applications, 3rd Edition,* John Wiley and Sons, NY, USA, **2022**.

5- Briggs, D.; Seah, M. P. *Practical Surface Analysis, Auger and X-Ray Photoelectron Spectroscopy*, *Vol. 1, 2nd Eds.*; Wiley, Chichester, **1996.**

6- Taner Camci, M.; Aydogan Gokturk, P.; Başaran, M.; Ulgut, B.; Kocabas, A.; Kocabas, C.; Suzer, S. Dynamics of Potential Screening upon Electrification of Solid-Ionic Liquid Interfaces Probed by XPS. In *Encyclopedia of Solid-Liquid Interfaces*; Elsevier, **2024**; pp 661–680. https://doi.org/10.1016/B978-0-323-85669-0.00097-0.

7- Selmi, G. S.; Lourenço Neto, E. R.; Lelis, G. C.; Okazaki, A. K.; Riul, A.; Braunger, M. L.; de Oliveira, R. F. Pulse Dynamics in Reduced Graphene Oxide Electrolyte-Gated Transistors: Charge Memory Effects and Mechanisms Governing the Ion-To-Electron Transduction. *Adv Electron Mater* **2025**, *11* (8). https://doi.org/10.1002/aelm.202400791.

8- Taner Camci, M.; Ulgut, B.; Kocabas, C.; Suzer, S. In Situ XPS Reveals Voltage Driven Asymmetric Ion Movement of an Ionic Liquid through the Pores of a Multilayer Graphene Electrode. *The Journal of Physical Chemistry C* **2018**, *122* (22), 11883–11889. https://doi.org/10.1021/acs.jpcc.8b02759.

9- Huang, M.-M.; Jiang, Y.; Sasisanker, P.; Driver, G. W.; Weingärtner, H. Static Relative Dielectric Permittivities of Ionic Liquids at 25 °C. *J Chem Eng Data* **2011**, *56* (4), 1494–1499.
